# Optimizing exciton transport in semiconductors

**DOI:** 10.1038/s41377-021-00657-9

**Published:** 2021-11-08

**Authors:** Jacob Embley, Xiaoqin Li

**Affiliations:** Department of Physics and Center for Complex Quantum Systems, Center for Dynamics and Control of Materials, The University of Texas at Austin, Austin, TX 78712 USA

**Keywords:** Photonic devices, Micro-optics

*eLight*, **1**, 6 (2021)

10.1186/s43593-021-00006-8

Excitons—charge-neutral quasiparticles comprised of bound electron hole pairs—can be formed within a wide range of semiconductors, including monolayer transition metal dichalcogenides (TMDs). The ongoing development towards practical excitonic devices necessitates a fundamental understanding of exciton transport in TMDs. Excitons in TMD monolayers exhibit ultrafast population relaxation times and correspondingly short diffusion lengths. In their article, Qi, Luo, and coworkers investigated how phonon scattering and disorder influence exciton diffusion in a WSe_2_ monolayer. They report optimized diffusion at an intermediate temperature between cryogenic and room temperatures. Most commendably, they combined results from three distinct optical techniques to support their hypothesis for this non-monotonic temperature dependence. Photoluminescence measurements suggested phonon scattering was the predominant impediment to transport at higher temperatures, while Raman characterization of micro strains indicated that a 2D disorder potential was the primary obstacle at lower temperatures. Pump probe experiments and associated theoretical modeling were performed to further corroborate the hypothesis. While open questions remain on exciton transport in TMD monolayers, new and exciting opportunities are emerging for controlling exciton transport in van der Waals heterostructures.

